# Supplementary material for: Biologically relevant laminin as chemically defined and fully human platform for human epidermal keratinocyte culture
Source: Nat Commun. 2018 Oct 30;9:4432. doi: 10.1038/s41467-018-06934-3 (PMC6207750; doi:10.1038/s41467-018-06934-3)
Supplement: Supplementary file 1 — Supplementary Information [file 41467_2018_6934_MOESM1_ESM.pdf]

# Biologically Relevant Laminin as Chemically Defined and Fully Human Platform for Human Epidermal Keratinocyte Culture

**Tjin et al.**

**Supplementary Figures:**

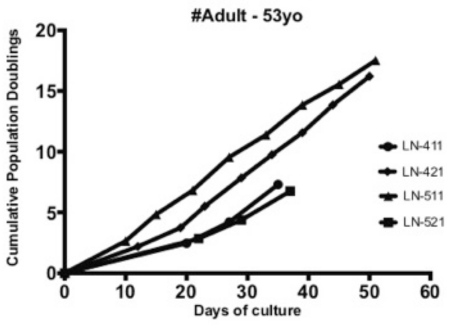


**Supplementary Figure 1.** Growth rate analysis between LN-411, LN-421, LN-511, and LN-521.

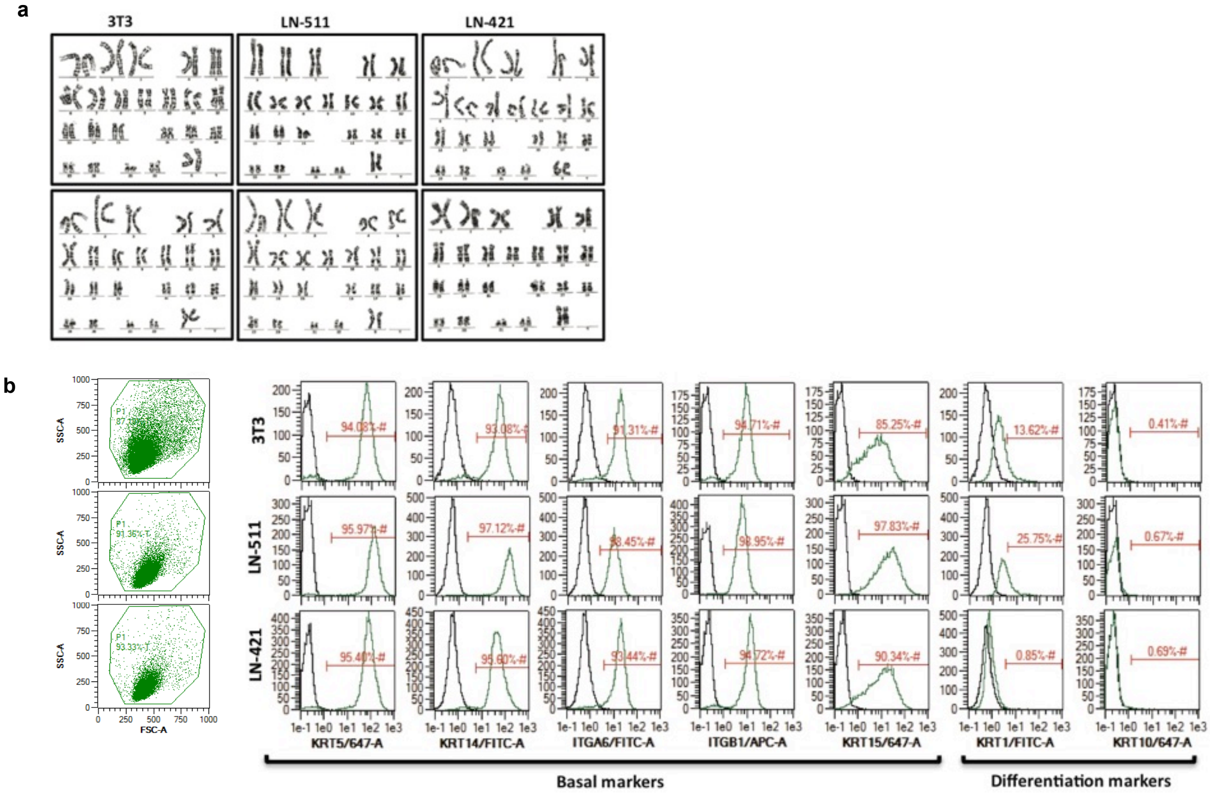


**Supplementary Figure 2 (a).** Karyotyping analysis of keratinocytes grown on LN-511, LN-421, vs on co-culture with 3T3. Keratinocytes in both passage 1 (top panels) and passage 9 (bottom panels) showed normal karyotyping with no observable translocations or chromosomal changes present. **(b).** Representative flow cytometry analyses of freshly isolated human epidermal keratinocyte markers expression after culturing on co-culture with 3T3-fibroblasts, LN-511, and LN-421.

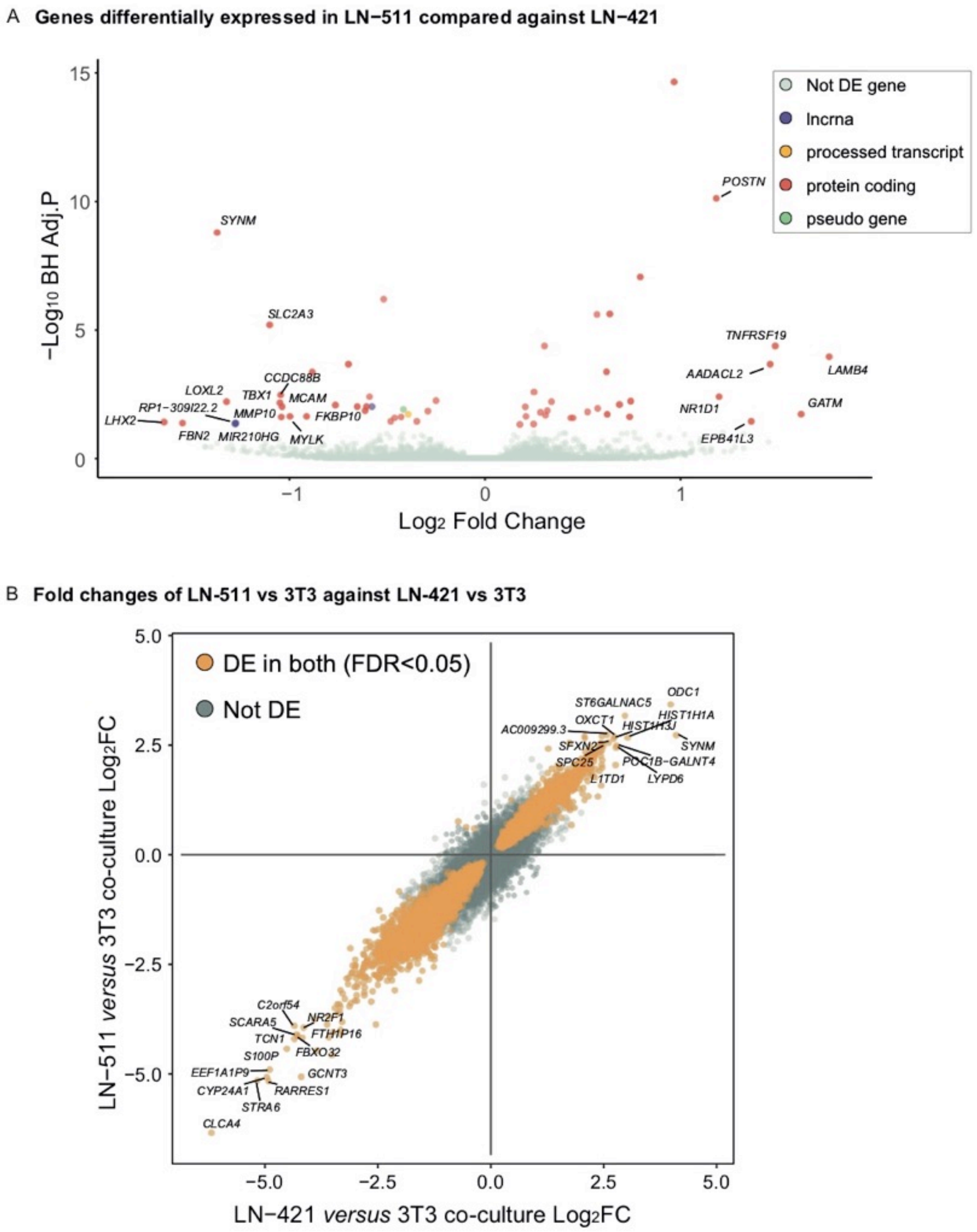


**Supplementary Figure 3 (a)**. Log2 gene expression fold change (Log2FC) of keratinocytes grown on two different laminin cultures (LN-511 and LN-421) both compared against keratinocytes grown on 3T3 cells. Every dot denotes a gene. Genes in orange are differentially expressed (DE) with both laminins (FDR < 0.05). Genes indicated in grey are not DE or DE in only one laminin substrate. Gene names are included for the top DE genes. (**b**). Volcano graph showing the differential expression results when comparing keratinocytes grown on LN-511 against the transcriptome of keratinocytes grown on LN-421. Gene names of top up and downregulated genes are shown. Genes that are not significantly differentially expressed are displayed in light green. DE genes (FDR <0.05) are colored by gene type.

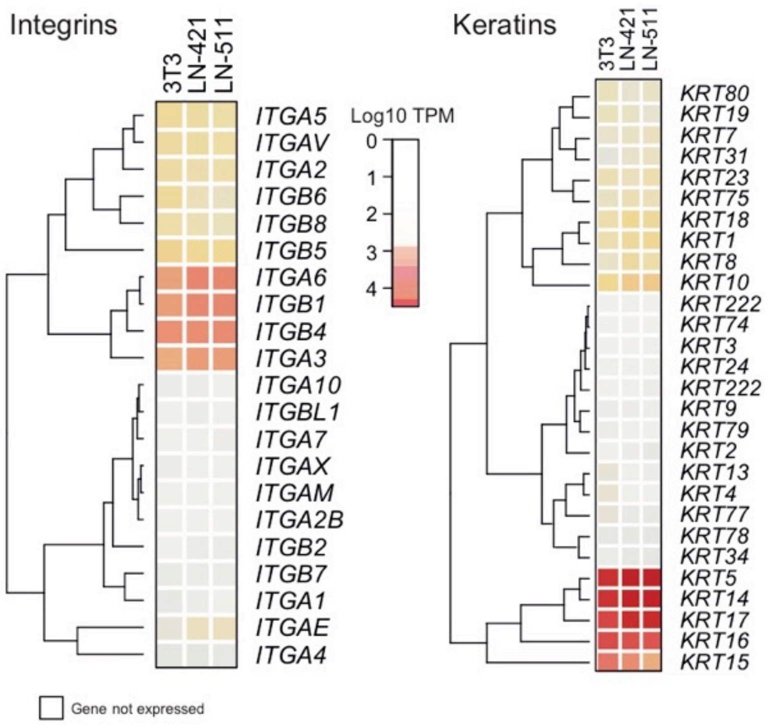


**Supplementary Figure 4.** Expression levels of integrin and keratin genes in keratinocytes growing on 3T3 co-culture, LN-421 and LN-511. Heatmap with transcript levels of integrin and keratin genes (averaged across biological replicates and represented as log10 of transcripts per million, TPM). Only genes with a total sum of more than one TPM across all samples are shown. Genes were hierarchically clustered using the “complete” method.

Supplementary Table 1. Skin donor information used in experiments

| Donor ID | Age | Gender | Body site |
|---|---|---|---|
| HEK 1 | 45 | F | abdomen |
| HEK 2 | 61 | M | abdomen |
| HEK 3 | 49 | F | breast |
| HEK 4 | 57 | F | breast |
| HEK 5 | 67 | F | abdomen |

**Supplementary Table 2. Primer sets used in this study, related to Fig. 1c and 2c.** All primers were used to detect the expression markers at different passages by qPCR. Sequences are given in the 5'-3' orientation.

| Gene | Forward Primer | Reverse Primer |
|---|---|---|
| *KRT5* | CAACCCACTAGTGCCTGGTT | GACACACTTGACTGGCGAGA |
| *KRT14* | AGCAGCAGAACCAGGAGTACAAG | GGCGGTAGGTGGCGATCT |
| *KRT1* | ATTTCTGAGCTGAATCGTGTGATC | CTGATGGACTGCTGCAAGTT |
| *KRT10* | ATGAGCTGACCCTGACCAAG | TCACATCACCAGRGGACACA |
| *LAMA1* | GTCAGCGACTCAGAGTGTTTG | AACTTGGGTGAAAGATCGTCAG |
| *LAMA2* | GAACCCGCAGTGTCGAATCT | GGGGAGTTAGCTGCCTTCA |
| *LAMA3* | CGTGAGGCTGAACTCCAAGT | CTGGATGTGGCTCCTTTGGT |
| *LAMA4* | GATGCCGTACTCTGCTGGTT | AGGCTGAGCTCAAAGCCATT |
| *LAMA5* | GGTGTGTCTCTGCGTGACAA | CCCCGACGTAGAAGACGAA |
| *LAMB1* | AGGAACCCGAGTTCAGCTAC | CACGTCGAGGTCACCGAAA |
| *LAMB2* | GCCCTGGGAACTTCGACTG | GGAAGCACTTCTTTTCGTCCTG |
| *LAMB3* | TCCTCTTGTGTTTTGCCCTG | CTGCCTGGAGTCACACTTG |
| *LAMB4* | CCTTGTGAATGTGACCCCGA | GGTTTGCACTGGTCGCATTT |
| *LAMC1* | GAGGCAAGATATCGCCGTGA | GTATCTCGCCTGTCCACTCG |
| *LAMC2* | CCAGGAGGGAAGTCTGTGATT | GCAGTGAATCCCATCAGTGTT |
| *LAMC3* | CTCTGCCTCAGAAGTCCCG | CTGGTGCAGCTTTGTGAGGG |
| *GAPDH* | AACAGCGACACCCACTCCTC | CATACCAGGAAATGAGCTTGACAA |

**Supplementary Table 3. List of antibodies used in this study, related to Fig. 1a, 2d-f, 4d-e, and Supplementary Figure 2b.**

| Antibodies | Source | Catalog number | Dilution |
|---|---|---|---|
| Alexa-Fluor 647-conjugated *KRT5** | Abcam | ab193895 | 1:400, 1:200* |
| FITC-conjugated *KRT14* (clone LL002)* | Abcam | ab77684 | 1:100, 1:100* |
| *KRT1* (clone LHK1)* | Abcam | ab81623 | 1:100, 1:100* |
| Alexa-Fluor 647-conjugated *KRT10** | Abcam | ab194231 | 1:100, 1:100* |
| *KRT15* (clone LHK15)* | Abcam | ab80522 | 1:100, 1:50* |
| *IVL* (clone SY5) | Abcam | ab68 | 1:100 |
| *FLG* | Abcam | ab81468 | 1:100 |
| FITC-conjugated CD49f* | BD Pharmingen | 555735 | 1:50, 20mL/1x106 cells* |
| APC-conjugated CD29* | BD Pharmingen | 559883 | 1:50, 20mL/1x106 cells* |
| p63 (clone 4A4) | Santa Cruz | sc-8431 | 1:100 |
| Alexa-Fluor 488-conjugated goat anti-mouse | Life Technologies | A11001 | 1:1000 |
| Alexa-Fluor 647-conjugated goat anti-rabbit | Life Technologies | A21245 | 1:1000 |
| Alexa-Fluor 488-conjugated goat anti-rabbit | Life Technologies | A11008 | 1:1000 |
| *LAMA1* | Lifespan Bioscience | LS-C25112 | 1:100 |
| *LAMA2* | Millipore | MAB1922 | 1:100 |
| *LAMA3* | Gift | BM165 | 1:40 |
| *LAMA4* | Abnova | MAB7869 | 1:100 |
| *LAMA5* | Abnova | H00003911-M01 | 1:200 |
| *LAMB1* | Abcam | ab44941 | 1:100 |
| *LAMB2* | Lifespan Bioscience | LS-C88433 | 1:100 |
| *LAMB3* | Santa Cruz | sc-20775 | 1:100 |
| *LAMC1* | Millipore | MAB1914P | 1:100 |
| *LAMC2* | Gift | P26 | 1:200 |
| *LAMC3* | Assay Biotech | C13074 | 1:25 |
| Alexa-Fluor 488-conjugated Ku80 | Abcam | ab198586 | 1:100 |

* used in FACS experiment
