# Supplementary material for: Biologically relevant laminin as chemically defined and fully human platform for human epidermal keratinocyte culture
Source: Nat Commun. 2018 Oct 30;9:4432. doi: 10.1038/s41467-018-06934-3 (PMC6207750; doi:10.1038/s41467-018-06934-3)
Supplement: Supplementary file 3 — Description of Additional Supplementary Files [file 41467_2018_6934_MOESM3_ESM.pdf]

Description of Additional Supplementary Files

File Name: Supplementary Data 1
Description: **Differential expression results.** Results included: Tab 1. Full list of differential expression results when comparing LN-421, LN-511 and 3T3. Log2 FoldChange, shrunken Log2-fold changes computed by DESeq2 package. BHadjP, Benjamini-Hochberg (BH) adjusted p-value. Tab 2: Functional gene set enrichment analysis (GSEA) results for the databases Hallmark in the RNA-seq differential expression analysis presented in tab1 (LN-421 and LN-511 both against 3T3). GSEA was computed ranking all genes by DESeq2 R package Wald test statistic. NES denotes normalized enrichment score (positive indicates upregulation whereas negative denotes downregulation). FDR denotes False Discovery Rate.

File Name: Supplementary Movie 1
Description: Keratinocytes single-cell migration pattern on LN-421.

File Name: Supplementary Movie 2
Description: Keratinocytes single-cell migration pattern on LN-511.

File Name: Supplementary Movie 3
Description: Colony migration pattern of keratinocytes on LN-421.
