# Supplementary material for: Biologically relevant laminin as chemically defined and fully human platform for human epidermal keratinocyte culture
Source: Nat Commun. 2018 Oct 30;9:4432. doi: 10.1038/s41467-018-06934-3 (PMC6207750; doi:10.1038/s41467-018-06934-3)
Supplement: Supplementary file 8 — Reporting Summary [file 41467_2018_6934_MOESM8_ESM.pdf]

nature research

Corresponding author(s): Karl Tryggvason
Alvin Wen Choong Chua

Reporting Summary

Nature Research wishes to improve the reproducibility of the work that we publish. This form provides structure for consistency and transparency in reporting. For further information on Nature Research policies, see Authors & Referees and the Editorial Policy Checklist.

Statistical parameters

When statistical analyses are reported, confirm that the following items are present in the relevant location (e.g. figure legend, table legend, main text, or Methods section).

n/a	Confirmed	
☐	☒	The exact sample size (n) for each experimental group/condition, given as a discrete number and unit of measurement
☐	☒	An indication of whether measurements were taken from distinct samples or whether the same sample was measured repeatedly
☐	☒	The statistical test(s) used AND whether they are one- or two-sided<br>*Only common tests should be described solely by name; describe more complex techniques in the Methods section.*
☐	☒	A description of all covariates tested
☐	☒	A description of any assumptions or corrections, such as tests of normality and adjustment for multiple comparisons
☐	☒	A full description of the statistics including central tendency (e.g. means) or other basic estimates (e.g. regression coefficient) AND variation (e.g. standard deviation) or associated estimates of uncertainty (e.g. confidence intervals)
☐	☒	For null hypothesis testing, the test statistic (e.g. F, t, r) with confidence intervals, effect sizes, degrees of freedom and P value noted<br>*Give P values as exact values whenever suitable.*
☒	☐	For Bayesian analysis, information on the choice of priors and Markov chain Monte Carlo settings
☒	☐	For hierarchical and complex designs, identification of the appropriate level for tests and full reporting of outcomes
☒	☐	Estimates of effect sizes (e.g. Cohen's d, Pearson's r), indicating how they were calculated
☐	☒	Clearly defined error bars<br>*State explicitly what error bars represent (e.g. SD, SE, CI)*

Our web collection on statistics for biologists may be useful.

Software and code

Policy information about availability of computer code

Data collection	BioRad CFX96 C1000 Real Time System (real time PCR), Leica LAS X (Imaging), Miltenyi Biotec MACSQuantify (Flow cytometry), Gene annotation was retrieved with R package biomaRt 2.30.0. Graphs were plotted with R package ggplot2 3.0.0 and assembled with Adobe Illustrator CC 2015.3.
Data analysis	MACSQuantify (Miltenyi Biotec) software Version 2.10 was used to analyse FACS data. Graphpad PRISM v7.0a was used for performing statistical analysis and generating figures. ImageJ/FIJI and Adobe Illustrator were used for imaging analysis and image generation. RNA-seq data processing done with STAR 2.5.2b (https://github.com/alexdobin/STAR), RSEM 1.2.31 (https://github.com/deweylab/RSEM/releases) and R packages DESeq2 1.14.1 and limma 3.30.13. RNA-seq data was analyzed with R package DESeq2 1.14.1 and GSEA 2-2.2.2 software (http://software.broadinstitute.org/gsea/index.jsp).

For manuscripts utilizing custom algorithms or software that are central to the research but not yet described in published literature, software must be made available to editors/reviewers upon request. We strongly encourage code deposition in a community repository (e.g. GitHub). See the Nature Research guidelines for submitting code & software for further information.

Data

Policy information about availability of data

All manuscripts must include a data availability statement. This statement should provide the following information, where applicable:

- Accession codes, unique identifiers, or web links for publicly available datasets
- A list of figures that have associated raw data
- A description of any restrictions on data availability

All relevant data that support the findings of this study are available from the corresponding author upon reasonable request. RNA-seq data is deposited in NCBI's Gene Expression Omnibus (GSE109645)

Field-specific reporting

Please select the best fit for your research. If you are not sure, read the appropriate sections before making your selection.

☒ Life sciences ☐ Behavioural & social sciences ☐ Ecological, evolutionary & environmental sciences

For a reference copy of the document with all sections, see nature.com/authors/policies/ReportingSummary-flat.pdf

Life sciences study design

All studies must disclose on these points even when the disclosure is negative.

Sample size	Sample size for each experiment is indicated in the figure legends. The sample size was chosen based on previous experience in the lab for each experiment to yield high power to detect specific effects. No statistical methods were used to predetermine sample size.
Data exclusions	No data were excluded.
Replication	All experimental findings were reproduced in multiple independent experiments as biological replicates. Data shown in figure panels are the mean of all independent biological repeats. The number of biological replicates is indicated in the figure legends.
Randomization	No formal randomization techniques were used.
Blinding	Blinding is not performed as the results for animal experiments are deterministic and visible when evaluated.

Reporting for specific materials, systems and methods

Materials & experimental systems

n/a	Involved in the study	
☒	☐	Unique biological materials
☐	☒	Antibodies
☐	☒	Eukaryotic cell lines
☒	☐	Palaeontology
☐	☒	Animals and other organisms
☐	☒	Human research participants

Methods

n/a	Involved in the study	
☒	☐	ChIP-seq
☐	☒	Flow cytometry
☒	☐	MRI-based neuroimaging

Antibodies

Antibodies used	Alexa-Fluor 647-conjugated KRT5 (Abcam, ab193895, 1:400); FITC-conjugated KRT14 (clone LL002) (Abcam, ab77684, 1:100); KRT1 (clone LHK1) (Abcam, ab81623, 1:100); Alexa-Fluor 647-conjugated KRT10 (Abcam, ab194231, 1:100); KRT15 (clone LHK15) (Abcam, ab80522, 1:100); IVL (clone SY5) (Abcam, ab68, 1:100); FLG (Abcam, ab81468, 1:100); FITC-conjugated CD49f (BD Pharmingen, 555735, 1:50); APC-conjugated CD29 (BD Pharmingen, 559883, 1:50); p63 (clone 4A4) (Santa Cruz, sc-8431, 1:100); Alexa-Fluor 488-conjugated goat anti-mouse (Life Technologies, A11001, 1:1000); Alexa-Fluor 647-conjugated goat anti-rabbit (Life Technologies, A21245, 1:1000); Alexa-Fluor 488-conjugated goat anti-rabbit (Life Technologies, A11008, 1:1000); LAMA1 (Lifespan Bioscience, LS-C25112, 1:100); LAMA2 (Millipore, MAB1922, 1:100); LAMA3 (Gift from Manuel Patarroyo's lab, BM165, 1:40); LAMA4 (Abnova, MAB7869, 1:100); LAMA5 (Abnova, H00003911-M01, 1:200); LAMB1 (Abcam, ab44941, 1:100); LAMB2 (Lifespan Bioscience, LS-C88433, 1:100); LAMB3 (Santa Cruz, sc-20775, 1:100); LAMC1 (Millipore, MAB1914P, 1:100); LAMC2 (Karl Tryggvason's laboratory in University of Oulu, Finland, P26, 1:200); LAMC3 (Assay Biotech, C13074, 1:25); Alexa-Fluor 488-conjugated Ku80 (Abcam, ab198586, 1:100)

Validation	Validation statements on specificity of the antibodies and citations can be found from manufacturers validation statements. Additionally also tested in the lab using appropriate positive and negative controls.

Eukaryotic cell lines

Policy information about cell lines

Cell line source(s)	Human primary cells were isolated from surgical waste from plastic surgery operations of healthy subjects with informed consent from these donors and ethics approval from SingHealth Centralised Institutional Review Board (CIRB). 3T3-J2 cells were gift from late Howard Green's laboratory.
Authentication	3T3-J2 were gift from late Howard Green's laboratory. All other cells used without any cell line authentication.
Mycoplasma contamination	MycoAlert mycoplasma detection kit [Lonza] is routinely used for mycoplasma testing and tested negative.
Commonly misidentified lines (See ICLAC register)	N/A

Animals and other organisms

Policy information about studies involving animals; ARRIVE guidelines recommended for reporting animal research

Laboratory animals	Female BALB/c nu/nu mice were purchased from Animal Resource Centre (ARC, Perth, Western Australia)
Wild animals	N/A
Field-collected samples	N/A

Human research participants

Policy information about studies involving human research participants

Population characteristics	Human primary cells were isolated from surgical waste from plastic surgery operations of healthy subjects. Information on age, gender, and sex are available in supporting information
Recruitment	All samples from subjects included in the study are taken with informed consent.

Flow Cytometry

Plots

Confirm that:

☒ The axis labels state the marker and fluorochrome used (e.g. CD4-FITC).

☒ The axis scales are clearly visible. Include numbers along axes only for bottom left plot of group (a 'group' is an analysis of identical markers).

☒ All plots are contour plots with outliers or pseudocolor plots.

☒ A numerical value for number of cells or percentage (with statistics) is provided.

Methodology

Sample preparation	Samples were harvested from cultured keratinocytes on Laminins or from 3T3 co-culture system and stained for respective biomarkers according to protocol described in materials method.
Instrument	Miltenyi Biotec MACSQuantify
Software	MACSQuantify (Miltenyi Biotec) Version 2.10
Cell population abundance	N/A
Gating strategy	Gating was done to separate debris and doublet cells using FSC and SSC. Threshold for positive-negative was defined at the max value of negative samples

☒ Tick this box to confirm that a figure exemplifying the gating strategy is provided in the Supplementary Information.
